# Exploring video recognition models for force estimation in small bowel surgical retractions

**DOI:** 10.1007/s11548-026-03583-6

**Published:** 2026-03-10

**Authors:** Kevin Wang, Ariel Rodriguez, Micha Pfeiffer, Sebastian Bodenstedt, Rayan Younis, Martin Wagner, Stefanie Speidel

**Affiliations:** 1 Translational Surgical Oncology, National Center for Tumor Diseases, 01307 Dresden, Germany; 2 German Cancer Research Center (DKFZ), Heidelberg, Germany; 3 Helmholtz-Zentrum Dresden-Rossendorf (HZDR), Dresden, Germany; 4 Faculty of Medicine and University Hospital Carl Gustav Carus, TUD, 01307 Dresden, Germany; 5 Centre for Tactile Internet with Human-in-the-Loop (CeTI), TUD, 01069 Dresden, Germany; 6 BMFTR Research Hub 6G-Life, TUD, 01069 Dresden, Germany; 7 Department of Visceral, Thoracic and Vascular Surgery, Faculty of Medicine and University Hospital Carl Gustav Carus, TUD, 01307 Dresden, Germany

**Keywords:** Laparoscopic surgery, Robot assisted surgery, Haptics, Computer vision

## Abstract

****Purpose**:**

Excessive retraction force during a minimally invasive surgery could cause tissue damage, affecting the surgical outcome. The integration of surgical force feedback relies on incorporating force sensors into the instruments, predominantly for robot-assisted surgery. However, such hardware integration is costly and challenging, and such instruments for conventional laparoscopic surgery are not yet widely adopted. We propose to use video as the primary source to objectively estimate tissue retraction force, which presents a promising approach to assess the skill of surgeons while retracting bowel tissue and addressing the current haptic deficiency without the need for special instruments.

****Methods**:**

We first introduce an experimental setup for the acquisition of a force-vision dataset with conventional laparoscopic instruments retracting a silicone small bowel phantom as an example. A novel data collection procedure is described along with the corresponding force-signal preprocessing methods. Then, we explore the performance of state-of-the-art ResNet and transformer-based computer vision models for force estimation. Two experiments are undertaken to assess feasibility of vision-based force estimation models.

****Results**:**

Results show that the models generalize across the collected dataset with varying phantom geometries and camera angles. We find ResNet-based models outperforming transformers, with all results matching or surpassing previous works. We further show that all models could perform real-time force estimation with respect to our data collection rate, with 3D ResNet being the fastest.

****Conclusion**:**

Vision-based force estimation based on the laparoscopic video feed is a promising way toward force estimation of tissue retraction. A novel use of computer vision models has been proposed with a low-cost data collection pipeline. Since the proposed clinical usage does not require customized equipment, seamless integration to the existing surgical framework is possible. The experimental results additionally demonstrate potentials in the approach to pave the way for real-time haptic feedback and quantitative skill assessment.

## Introduction

Gentle handling of tissues is considered to be the first fundamental principle of surgical skill and practice [1]. This holds true as excessive retraction force can lead to trauma in many organs, adversely affecting the quality of surgery [2], which in turn negatively influences postoperative outcomes of patients [3, 4]. In gastrointestinal surgery, the small intestine is often manipulated to provide better access to the operating field, but it is particularly vulnerable to injury from excessive retraction [5, 6]. It should be noted that small bowel retraction is commonly performed during various gastrointestinal laparoscopic cancer surgeries, such as ileocolostomy, to facilitate the lifting and dissection of the bowel under traction or to expose adjacent anatomical structures obscured by the bowel. At the beginning of one such surgery, bowel retraction often forms part of the exploratory phase, as the small intestine frequently overlies other critical anatomical regions. In non-oncological procedures, manipulation of the small bowel is also common, particularly during laparoscopic management of small bowel obstructions.

The minimally invasive technique is often used to perform gastrointestinal surgery [7], with conventional laparoscopic surgery being significantly more widespread than robot-assisted surgery (RAS). It consists of inserting thin rod-shaped instruments into the abdomen, but the limited force feedback provided by the instruments in conventional laparoscopic surgery, as well as the complete loss of force feedback in RAS, makes it challenging to prevent excessive retraction force [8]. Since the introduction of the da Vinci 5 Surgical System (Intuitive Inc., Sunnyvale, USA) with force sensors, the measurement of retraction force and even real-time force feedback are now possible for RAS. However, this benefit is still unavailable for the significantly more widespread conventional laparoscopic surgery and present RAS systems without custom-made mechanical sensors [9, 10]. In the meanwhile, it has been shown that experienced surgeons can deduce the force applied by their laparoscopic instruments using only visual cues [11], also referred to as ‘visual-haptics.’ As such, gentle tissue retraction in laparoscopic surgery is a skill that comes with experience [12, 13].

Thus, the measurement of retraction force after surgery could be helpful to assess surgical quality and to inform novice surgeons to improve their surgical skill [14]. However, it remains a challenge to quantify retraction force of the small bowel during laparoscopic procedures on a large scale. Popular surgical skill assessment tools include tissue handling as a component, but it remains a subjective measure [15, 16].

Following the approach of previous works leveraging computer vision (CV) on force feedback for RAS [17, 18], our CV methods—similar to surgeons using ‘visual-haptics’—aim to address this deficiency—especially for laparoscopic procedures. By adopting vision-based force estimation models, we eliminate difficulties surrounding mechanical sensors. Hence, our methods could be applied to both RAS and laparoscopic surgery in a scalable manner, where we objectively estimate force using video input only. Those force estimations could then be used for personalized postoperative surgical quality assessment for surgeons.

To train and validate vision-based force estimation models, real-world data on retraction force applied during laparoscopic surgery on small intestines are needed but very limited [19–21]. Though some datasets using solid silicone phantoms and ad hoc animal tissue are available in the context of surgical palpation and retraction [18, 22], matching force-video dataset for the intestines is still lacking. Within the scope of this study, it must be noted that datasets captured using solid block phantoms may not provide similar mechanical responses to the intestines. Considering the easily deformable, thin-walled, and hollow-lumen structure of the small intestine, there will be a large domain gap if CV models are trained on block phantoms then directly translated to our context without thorough fine-tuning on intestinal data. Thus, effective real-world data collection and the development of data-efficient algorithms with generalization capabilities are paramount for this emerging field.

In this paper, our contributions are:

- a data collection pipeline agnostic to the surgical setting and technique, capturing videos from different camera angles with randomized pulling motions to enhance dataset variability,
- a video-force dataset with silicone small intestine phantom to enable model training—where it could be used as benchmark or pretraining data for future research,
- successful generalization capabilities of vision-based force estimation models on a preliminary dataset, evaluating their performance on unseen camera perspectives and varying bowel geometries from limited data.

All data, hardware design, and code are available upon publication here: https://gitlab.com/nct_tso_public/smallintestine-force-estimation.

**Fig. 1:**
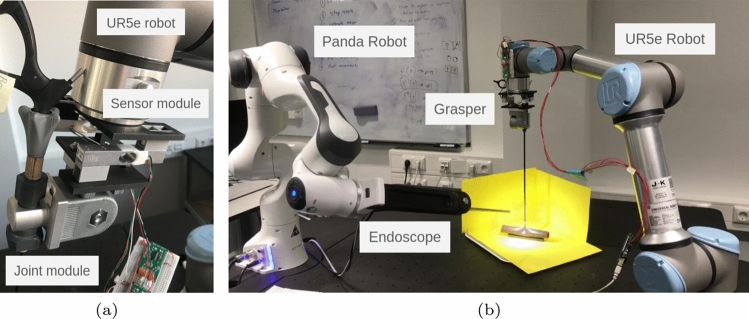
Experimental setup in detail. **a** Mechanical design of the sensor module and the grasper joint. **b** Full setup with background and a phantom

### Related works

Vision-based force estimation methods are less common for laparoscopy than for RAS. This is mainly due to the absence of precise instrument position tracking and robotic state data to measure force in conventional laparoscopy. Previous research has broadly divided these methods into interpolation-based, learning-based methods and mixed-approach methods [23]. Interpolation-based methods capture and track surface features for geometry reconstruction [24]. These models usually involve calculations with prescribed physical models [25], pre-measured deformation-force relationships [26, 27], or finite-element analysis [28]. However, they do not take computational cost into account, despite computational hardware performance being critical for real-time clinical use. Learning-based methods do not perform any physics simulations but solely rely on a dataset with synchronized video and force signal—preferably with robot state data [29]. It has been shown that inclusion of state information increases estimation accuracy and improves generalizability when faced with unknown tools and materials [18]. Furthermore, the absence of material property input does not decrease estimation results [30]. Compared to interpolation-based methods, learning-based methods are better suited for intraoperative tasks by avoiding specifically solving the deformation problem. Generalizability is still an overall concern due to limited data realism and diversity. Mixed-approach methods amalgamate the previous categories by using CV to reconstruct deformation followed by machine learning force estimation methods. The surface reconstruction usually consists of creating point clouds and encoding temporal information with neural networks or filter blocks [17]. Unlike interpolation-based and mixed methods that rely on explicit surface reconstruction, we predict force directly from monocular video segments using vision models originally intended for other natural image applications. We compare the performance of a vision transformer and ResNet architectures, evaluating their ability to predict pulling force without requiring material properties or robotic state data.

## Method

Section 2.1 describes the hardware setup including the phantom, the robots, and the sensors. Section 2.2 describes how video and force data are procured and synchronized, followed by implementation details on the CV models in Sect. 2.3. A description of experiments is presented in Sect. 2.4.

### Experimental setup

To generate data for vision-based force estimation, we aim to mimic a laparoscopic setting, including the interaction between surgical instrument and phantom bowel through pulling motions. To achieve precise and controlled force measurements, we use a robotic arm for controlling the instruments, in order to accurately regulate the pulling distance and applied force.

For data capturing, a background is constructed by perpendicularly joining three large pieces of yellow fat-colored cardboard. A silicone bowel phantom (Kroton Medical Technology, Warsaw, Poland) is positioned centrally in front of the background.

The robot assembly is composed of an UR5e robot arm (Universal Robots, Odense, Denmark), a force-sensor module, and a standard laparoscopic surgical grasping instrument (Aesculap AG, Tuttlingen, Germany) secured in a joint module. We opt for a standard, non-wristed surgical instrument to collect data specifically for conventional laparoscopic surgery, as this is the most common technique for minimally invasive gastrointestinal surgery [7], but our data collection method is agnostic to the chosen surgical technique, organ, and instrument. Additionally, the method and setup can also be used for in general haptic applications, such as in [31]. The joint module involves two lockable halves and ensures stability while allowing the instrument to be angled (Fig. 1a). The joint module is attached to the force-sensor module, which is then fixed to the robot end-effector (Fig. 1b). Redesigned from the setup of [32], the force-sensor module consists of two TAL220 load cells (HT Sensor Technology, Xi’an, China) mounted on robust 3D-printed casings. The load cells are secured diagonally to ensure the measurement axis is aligned with the movements. The force signals are collected via two HX711 amplifiers and an Arduino microcontroller (BCMI Labs SA, Chiasso, Switzerland) at 11 Hz in considerations for reduced noise.

The robot arm is controlled via a Robot Operating System (ROS) controller publishing its state at 500 Hz. A ROS topic communicates the pulling translation of the arm along with defined movement durations. Meanwhile, force data from the Arduino are published to another ROS topic. The video data are captured with a TIPCAM1 S 3D endoscope (Karl Storz SE & Co. KG, Tuttlingen, Germany) mounted on a Panda robot arm (Franka Robotics GmbH, Munich, Germany) for ease of adjustments between, and stability through experiments. Although we recognize that stereo vision is available in most surgical robotic systems and studies [17, 33], we still choose monocular vision as input to the CV model as it is more widely used in conventional laparoscopic surgery, especially considering the cost, additional setup, discomfort associated with 3D glasses, and limited rotational capabilities of angled stereo endoscopes. Hence, we have captured monocular video data to reflect the more widespread use of 2D video compared to 3D video [34]. Output images are published in a separate topic with a resolution of 960x540 pixels at 30 Hz. Since our force estimation models only require monocular video as input, only the right camera frame is used. All data are saved with time stamps.

Since the force signal is published at a different frequency than the video, all ROS topics are temporally aligned with force-signal time stamps serving as the reference. The closest video frame is selected based on the time stamps of the data—resulting in videos approximately 130 frames in length after synchronization.

### Data collection and preprocessing

We start the data collection by positioning the UR5e robotic arm at a predefined initial state. At the start of each trial, the robot pivots at the grasping point by adjusting its pitch and yaw angles. The pivot is essential to ensure that each video captures pulling motions in a different direction. The pitch angle is randomly selected from a range of $-15^\circ$ and $1.71^\circ$, while the yaw angle is sampled within $-1.71^\circ$ and $15^\circ$. These specific angle constraints were determined based on the physical setup of the cardboards, where exceeding these limits could cause collisions. By restricting the pitch and yaw angles within these ranges, we maintain safe and controlled movement while maximizing the available motion space. It should also be noted that the grasper has never obscured the phantom in pulling motions, nor has any retraction taken place along the depth axis of the endoscope, leading to visual ambiguities. After this process, the load cells are zeroed to eliminate environmental force and moments. Then, the video recording starts.

A pulling distance and a movement duration are randomly chosen within their limits. The pulling distance constraints are 3 mm and 2 cm. On the other hand, the movement duration is sampled between 1 to 2 s. Given the selected parameters, the robot then executes the pulling motion accordingly. Upon reaching the target position, the system remains stationary for a sampled duration between 0.1 and 0.5 s before proceeding to the next movement. The maximum pulling force that is recorded corresponds to approximately 5 N.

**Fig. 2:**
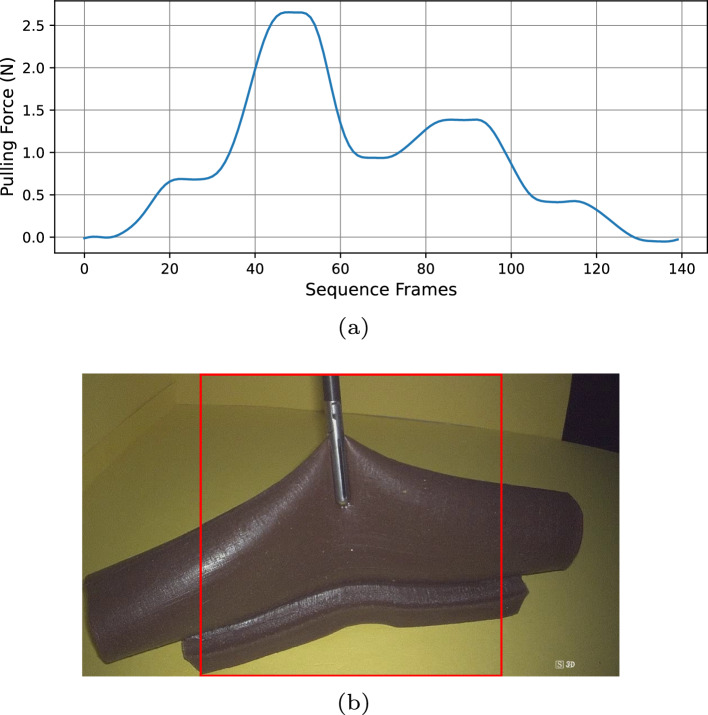
Force and video collections. **a** Pulling force trajectory of a recorded video. **b** Exemplary frame of a recorded video. The red square shows the applied crop

The above process is repeated five times in each recording. In each iteration, a new pulling distance and movement time combination is sampled while keeping the pitch and yaw angles fixed, introducing variability into the dataset. After completing the last pulling motion, the robot goes back to the position where no pulling force is applied. An example of a force trajectory of one recorded video can be seen in Fig. 2a.

Before the data can be passed to the network, we define the pulling motion to be of the positive force direction, inverting any force signals otherwise. The net pulling force is therefore the sum of the two load cell readings. Due to isolated large erroneous signal spiking, we cut off values at $-2$ and 10 N, as we observe normal recorded signal trajectory values should never be outside this range, taking into account that noisy trajectories may situate beyond 0 and 5 N. Any value beyond the cutoff lines is revised to the average of the previous and the following readings. The same correction is applied when a force reading is found to be more than 1.5 times the value of its previous counterpart—stemming from the knowledge that no defined robotic movements in the experiments could lead to such drastic accelerations.

After removing improbable sensor readings, the signals undergo fast Fourier transform and a cutoff of 0.15 cycles/frame is defined. The transformed spectrum above 0.15 or below $-0.15$ cycles/frame is nullified to remove high-frequency noise. At last, the inverse transformation produces the filtered signal.

In all, a dataset of 50 videos has been recorded. The videos are cropped into $540\times 540$ pixels before resized to 112x112 pixels. A clear view of the grasper is guaranteed when manually positioning endoscope. Each video is given a random camera position, but the grasper is centrally positioned at the start of each video and its motions are in view at all times. The endoscope is always at an oblique angle to the grasper, resembling the instrument setup in a standard minimally invasive surgery with minimal visual ambiguity. It should also be noted that the camera angles ensure only the constructed background, the grasper, and the phantom are visible after data processing, as shown in Fig. 2b. Additionally, only one pulling direction is represented per video. The dataset is divided into sets of ten recordings with each set corresponding to one bowel geometry. The five bowel geometries include a straight bowel and four bent counterparts with randomized angles between within $30^\circ$ ($6^\circ$, $16^\circ$, $21^\circ$, $25^\circ$), maintaining a circular lumen along the length of the phantom.

### Network architectures and training

Three vision-based force estimation architectures are trained: 3D-ResNet-18, $(2+1)$D ResNet [35], and Video Swin Transformer [36], with 3D-ResNet-18 serving as the baseline model. A dataloader based on the design of [31] reads video data and force ground truth. A major advancement, thanks to the models to inherently process video data, is that once the data are loaded, a temporal window of defined length is read as a unit and rolls to later frames, eliminating the necessity of additional modules such as LSTM or RNN to capture temporal information [18].

We change the final fully connected layer of the models to produce a regression output instead of a classification output. The feature vector obtained from the vision models is first passed through a fully connected layer that reduces its dimensionality to 512 neurons, followed by the application of a ReLU activation function to introduce nonlinearity. Subsequently, a dropout rate of 0.5 is employed to prevent overfitting. Finally, the processed neurons are input into a fully connected layer that outputs a one-dimensional vector corresponding to the predicted force. Training the three models using mean-square-error (MSE) loss and a batch size of 5 for 50 epochs shows that the best learning rate is $10^{-4}$. We train the models using mixed precision on one Nvidia RTX A5000 GPU.

**Fig. 3:**
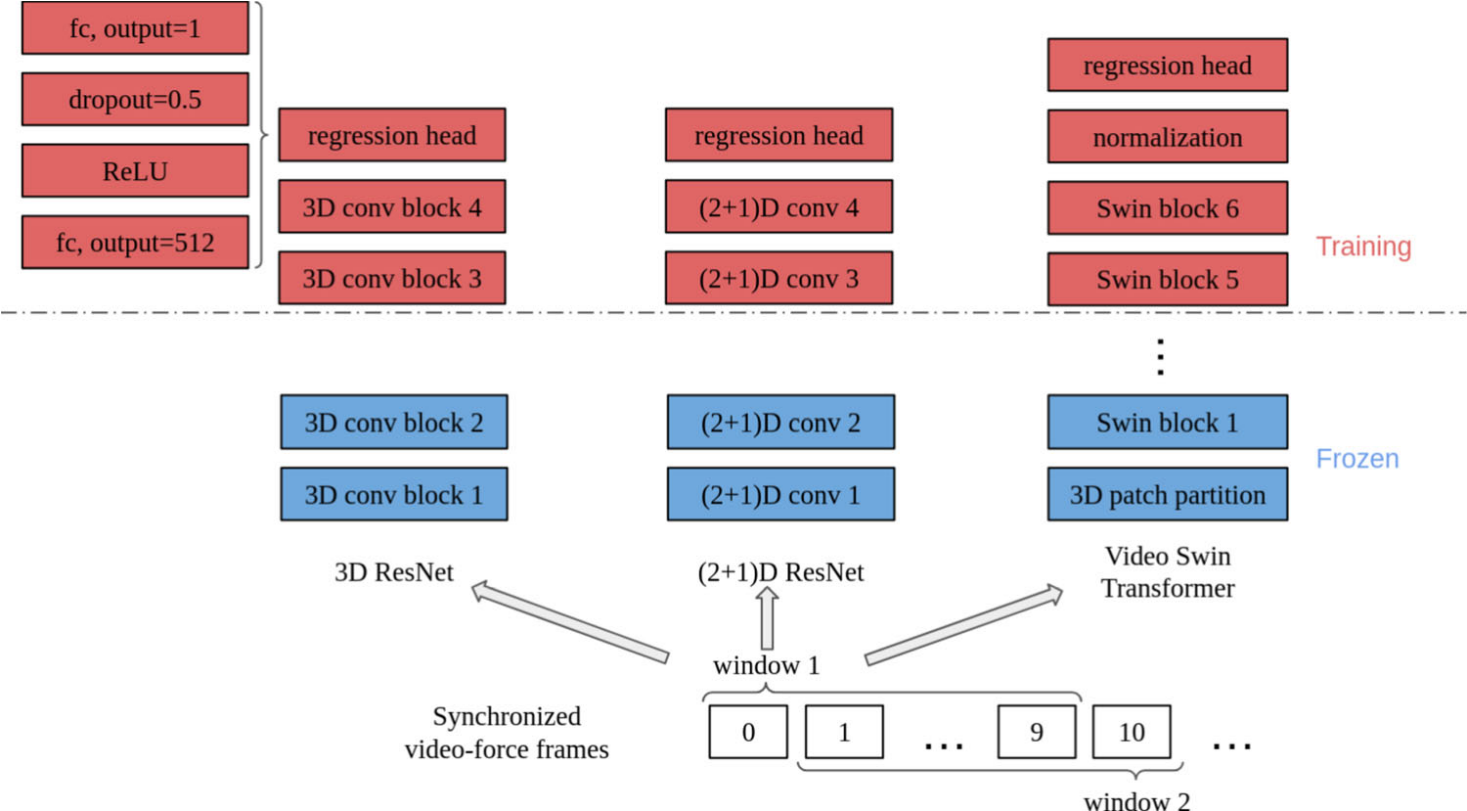
Data loading and training: the rolling window method is illustrated with assumption that window length is set to 10 frames. Each loaded window is then passed into a model for training, where the first blocks are frozen and only the upper feature blocks and regressions heads are trained

For the three models, we initialize training using pre-trained weights from the Kinetics-400 dataset [37]. In the interest of memory requirements and generalizability, we freeze the initial layers during training. In the case of the 3D-ResNet-18 and $(2+1)$D, we fine-tune the third and fourth blocks and the regression head. For the transformer, we fine-tune the fifth and sixth feature blocks, the final normalization layer, and the regression head. To mitigate the risk of overfitting, we apply L2 regularization to the trainable weights of the model, using a weight decay equal to $10^{-2}$. The data loading and training regime described above is also illustrated in Fig. 3.

To encourage gradual convergence, we use cosine annealing, with a minimum value of $10^{-5}$ as the learning rate decay schedule with the AdamW optimizer, providing a stable fine-tuning process. During training, data augmentation, such as horizontal flip, Gaussian blur, and RGB shift, was applied to the input sequences, as we find the models exhibit tendencies to overfit in its absence, reducing generalizability.

### Experimental design

We design two fivefold cross-validation experiments, selecting root-mean-square error (RMSE) as evaluation metric. Additionally, normalized RMSE (NRMSE) is also reported with respect to the total range of applied forces in the test sets, as calculated in equation 1. Primary assessments in the generalizability of the models are undertaken in two key aspects: unseen phantom geometries and unseen camera angles. In both experiments, we keep a ratio of 60% of the data for training, 20% for validation, and 20% for testing.

1 $$\begin{aligned} \text {NRMSE} = \frac{ \sqrt{ \frac{1}{N} \sum _{t=1}^{N} \left( F_{\text {estimated},\,t} - F_{\text {measured},\,t} \right) ^2 } }{ F_{\max } - F_{\min } } \end{aligned}$$

**Table 1:** Test RMSE (N) and NRMSE for geometry experiments

Metric	Window length	3D ResNet	$(2+1)$D ResNet	Video Swin Transformer
RMSE (N)	10	**0**.**323** $\pm$ **0**.**066**	0.327 $\pm$ 0.068	0.574 $\pm$ 0.087
	20	0.342 $\pm$ 0.074	**0**.**339** $\pm$ **0**.**059**	0.469 $\pm$ 0.065
	30	0.374 $\pm$ 0.100	**0**.**340** $\pm$ **0**.**061**	0.468 $\pm$ 0.081
NRMSE	10	**0**.**077** $\pm$ **0**.**016**	0.078 $\pm$ 0.017	0.136 $\pm$ 0.023
	20	0.081 $\pm$ 0.017	**0**.**080** $\pm$ **0**.**014**	0.111 $\pm$ 0.015
	30	0.088 $\pm$ 0.019	**0**.**081** $\pm$ **0**.**014**	0.110 $\pm$ 0.014

Best results for each window length are highlighted in bold

**Table 2:** Test RMSE (N) and NRMSE for camera angle experiments

Metric	Window length	3D ResNet	$(2+1)$D ResNet	Video Swin Transformer
RMSE (N)	10	**0**.**318** $\pm$ **0**.**050**	0.331 $\pm$ 0.102	0.518 $\pm$ 0.059
	20	**0**.**313** $\pm$ **0**.**028**	0.332 $\pm$ 0.071	0.425 $\pm$ 0.050
	30	0.328 $\pm$ 0.057	**0**.**321** $\pm$ **0**.**033**	0.404 $\pm$ 0.064
NRMSE	10	**0**.**070** $\pm$ **0**.**011**	0.075 $\pm$ 0.025	0.115 $\pm$ 0.015
	20	**0**.**069** $\pm$ **0**.**007**	0.073 $\pm$ 0.015	0.094 $\pm$ 0.012
	30	0.072 $\pm$ 0.013	**0**.**071** $\pm$ **0**.**004**	0.089 $\pm$ 0.015

Best results for each window length are highlighted in bold

The first experiment addresses the challenge of unseen phantom geometries. In each fold of this experiment, one bowel geometry is completely excluded from training and used solely for testing. From the remaining four geometries, two videos per geometry are randomly selected for validation, while the remaining videos are used for training.

For the second experiment evaluating generalization to unseen camera angles, the data are split differently. As every video in the dataset has a unique camera angle, we select two videos from each of the five geometries for validation and two for testing, while the remaining six videos are used for training. In the first fold, videos 1 and 2 from each geometry are assigned to the test set, while videos 3 and 4 are used for validation. In the second fold, videos 3 and 4 are used for testing, while videos 5 and 6 are assigned to validation. This process continues in subsequent folds, ensuring that every video is eventually included in both validation and test sets across different iterations.

In the scope of the first and second experiments, we also report the inference time of every model with different window lengths. With this measurement, we show the inference speed capabilities of the methods.

To compare our method with existing CV models, we additionally conducted two benchmark experiments. In the third experiment, a dataset published by Chua et al, containing both retractions and palpations [18], is selected. In order to follow the evaluation techniques of the benchmark method, modifications are made where the last fully connected layer of each model has been replaced to output three values representing forces in *x*, *y*, *andz* directions. To maintain consistency with the benchmark, center cropping was performed at 300 $\times$ 300 pixels as specified in [18]. The cropped images were then resized to $112\times 112$ pixels to match the input dimensions of our models. All training hyperparameters were maintained consistent with those used for training on our dataset. The models were trained for 50 epochs with the same dataset divisions outlined in [18] subject to dataset completeness.

The fourth experiment, using the same model modifications specified in the last, is also found upon it. A more realistic dataset has been used, which contains manipulation recordings with raw chicken tissue instead of silicone setups [22]. In the benchmark experiment, images are cropped to windows of $234\times 234$ pixels, centered on the M2 keypoint. However, since our pipeline does not incorporate keypoint detection, we retain the 300 $\times$ 300 center crop. The model from the third experiment has been further fine-tuned on the realistic dataset for 50 epochs maintaining the exact same hyperparameters. The results from this experiment will be able to indicate the models’ generalizability to data from a different domain.

## Evaluation

**Fig. 4:**
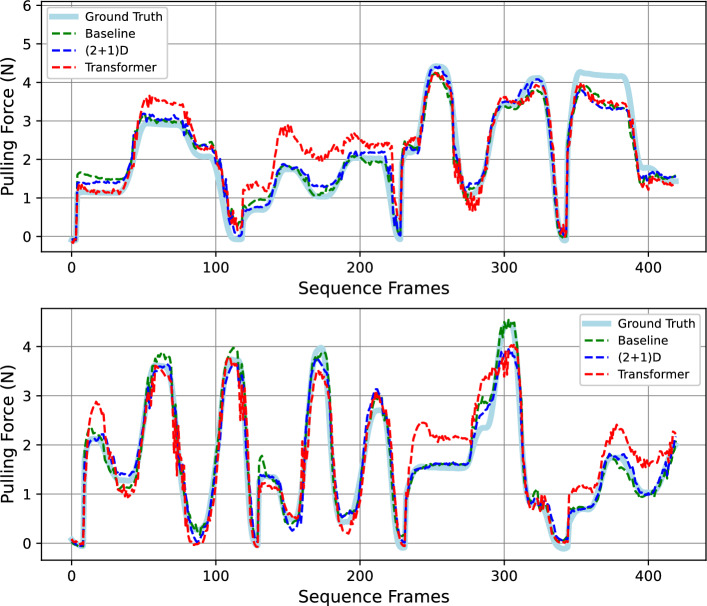
Two segments of predicted force trajectories from testing dataset with 3D and $(2+1)$D ResNets having higher accuracy compared to the transformer model

Throughout the first and second experiments, test results from Tables 1 and 2 show that the ResNet-based models are able to estimate applied force significantly more accurately than the Video Swin Transformer (paired *t* test, $p<0.0001$). However, the same test ($p=0.90$) also shows that $(2+1)$D ResNet demonstrates no superior performance than 3D ResNet, nor vice versa. We speculate that these findings are due to transformers being associated with worse performance than convolutional neural networks when training data are scarce, as also seen in the original Vision Transformer [38]. It is important to note at this point that training the full transformer model might improve its performance, with the trade-off of memory consumption and training-time. The trajectories in Fig. 4 illustrate this point where the transformer trajectory could follow the dynamics of the ground truth, but is often separated from the ground truth by a margin. Noting that standard deviations in Table 1 across experiments are generally only around 20%, we conclude that the stable results from cross-validation experiments indicate that all our models have performed well in the perspective of generalization over phantom geometry and camera angles. Thus, the main assessments of the experiments have been satisfied.

We ran further tests where we show that all three models are real-time capable, as Table 3 contains models’ inference time per output frame. In order to provide real-time visual force feedback, such as an overlay on the surgical video, the model update rate should match the video frame rate (larger than 25 frames per second) with system delay under 100ms for unhindered operations [39]. The results demonstrate that 3D ResNet stands as the fastest model, while Video Swin Transformer is the slowest. It is also confirmed that, independently of the model, with smaller window length the inference time is faster which it is an expected result. That said, the slowest condition of all scenarios offered 0.034 s per frame, corresponding to approximately 29Hz, which is above the data collection rate. Therefore, it can be concluded that all models can perform real-time inference with our setup.

This finding indicates that our method enables the development of systems for building real-time force feedback systems. In this case, additional hardware systems providing haptic feedback similar to the Da Vinci 5 system are further required. However, the setup cannot currently be implemented clinically without regulatory obstacles. We see, however, potential in providing visual force feedback in lieu, which can be deployed on a large scale.

While investigating whether varying input window lengths affect testing results using paired t tests, we find that using 10-frame windows leads to worse results than cases with 20-frame windows ($p=0.02$). However, models using 30-frame windows produce insignificant improvements ($p=0.73$) than results with 20-frame window inputs. Hence, we conclude that 20 frames is the ideal input sequence window length where enough temporal information has been included while not requiring additional memory.

**Table 3:** Inference time (secs) against window lengths

Model	10 frames	20 frames	30 frames
3D ResNet	**0**.**011**	**0**.**018**	**0**.**023**
$(2+1)$D ResNet	0.011	0.021	0.028
Video Swin Transformer	0.023	0.030	0.034

The bold values refer to best performance in each column

To compare our methods to others in the third and fourth experiments, we train our models with a window length of 20. Table 4 demonstrates results from the third experiment using silicone manipulation data. Since our model has only visual inputs due to considerations on clinical implementation, the benchmark RMSE values are chosen from the vision-input only models in the work of Chua et al. [18]. It is also shown in Table 4 that the ResNet-based networks provide for higher force estimation accuracy than the Video Swin Transformer and the referenced benchmark method in workspace and viewpoint configurations, as well as the unseen scenarios. Given such performance, it can be concluded that the ResNet-based methods should consistently perform better than the transformer and the benchmark method. Comparing UM (Unseen Material), UT (Unseen Tool), and mean configuration results from Table 4 further show that the models perform well to seen workspace and viewpoint configurations, but not to the two unseen cases, where they could lead to double the error. Therefore, complete retraining on a new dataset is advisable to achieve the best result when the test setup or material is different from that of training. Moreover, in considerations of generalization to increase accuracy in unseen environments, we suggest adding diversity to the data in terms of background, retraction/palpation, and surgical instrument.

**Table 4:** RMSE (N) across workspace and viewpoint configurations

Model	Workspace and viewpoint configuration											UM	UT
	L3	L2	L1	C	R1	R2	R3	Z1	Z2	Z3	Mean		
3D ResNet	0.421	**0**.**362**	0.362	0.435	0.318	0.347	0.418	**0**.**552**	0.455	0.501	0.417	**1**.**101**	0.774
$(2+1)$D ResNet	**0**.**407**	0.346	**0**.**334**	**0**.**417**	**0**.**293**	**0**.**340**	**0**.**404**	0.556	**0**.**448**	**0**.**491**	**0**.**404**	1.151	**0**.**727**
Video Swin Transformer	0.467	0.437	0.443	0.535	0.382	0.408	0.492	0.634	0.563	0.651	0.501	1.177	0.799
Chua et al	0.521	0.499	0.504	0.496	0.456	0.499	0.465	0.721	0.728	0.714	0.560	1.837	0.740

The bold values refer to best performance in each column

**Table 5:** Test NRMSE for fine-tuning on silicone and realistic dataset

Model	Silicone			Realistic		
	X	Y	Z	X	Y	Z
3D ResNet	0.062 $\pm$ 0.022	0.084 $\pm$ 0.025	0.053 $\pm$ 0.033	0.102 $\pm$ 0.025	**0**.**114** $\pm$ **0**.**034**	0.106 $\pm$ 0.033
$(2+1)$D ResNet	**0**.**061** $\pm$ **0**.**026**	**0**.**082** $\pm$ **0**.**024**	**0**.**050** $\pm$ **0**.**032**	0.101 $\pm$ 0.025	0.115 $\pm$ 0.030	0.106 $\pm$ 0.032
Video Swin Transformer	0.078 $\pm$ 0.024	0.091 $\pm$ 0.024	0.060 $\pm$ 0.031	0.105 $\pm$ 0.023	0.121 $\pm$ 0.034	0.108 $\pm$ 0.026
GNN [22]	0.115 $\pm$ 0.014	0.126 $\pm$ 0.015	0.093 $\pm$ 0.022	**0**.**085** $\pm$ **0**.**014**	0.130 $\pm$ 0.025	0.108 $\pm$ 0.030
FCN [22]	0.116 $\pm$ 0.014	0.121 $\pm$ 0.015	0.093 $\pm$ 0.026	**0**.**085** $\pm$ **0**.**015**	0.127 $\pm$ 0.021	**0**.**103** $\pm$ **0**.**024**

The bold values refer to best performance in each column

To examine how our models perform with complex background, Table 5 demonstrates results from the fourth experiment with the realistic dataset using chicken tissue. We benchmark our models with the graph neural network (GNN) and fully connected neural network (FCN) trained by [22]. It is shown that our models outperform the referenced GNN and FCN methods in the silicone dataset and in the Y-axis of the realistic dataset, but not in the X- and Z-axis—nevertheless—the differences appear small. We infer that this result is due to the knowledge of the past that our models retain from the window input approach, which the benchmarks lack. We notice that model performance degrades significantly when using the realistic dataset. It indicates that data complexity is a factor of estimation accuracy, and it may not be wholly compensated by retraining or fine-tuning. We speculate that adding other input modalities, such as robot state, might improve the results [18].

## Conclusion

This work contributes to advancing scalable surgical quality assessment by demonstrating that computer vision can serve as a reliable surrogate for direct force sensing in tissue manipulation. We introduce the use of video as a primary modality for objectively estimating tissue retraction force, a novel approach that addresses the lack of haptic feedback in minimally invasive surgery. A novel low-cost robotic setup has been presented to collect retraction data for training CV models. Additionally, a data collection pipeline, agnostic to surgical setting and operating technique, led to a dataset with videos from different camera angles with randomized pulling motions. We then demonstrated successful generalization capabilities of the vision-based force estimation models on the dataset, showing small estimation errors and real-time inference capabilities.

Comparing the RMSE values with previous force estimation works, we find that our errors are comparable to or lower than those reported in the literature. However, direct comparison in the case of small bowel is unavailable—and for this reason we will release our dataset. Results show that our models are able to estimate pulling force with the phantom—which is more fragile and deformable than solid silicone phantoms previously used [30]. It should be further noted that our models use vision alone, without referring to depth estimation [40], robotic state [18], or physical simulations [28]. This design feature reflects our considerations in that tracking non-robotic laparoscopic tools is very technically demanding and measured positions are usually not precise enough to support force estimation. Hence, out methods remove the dependency on positional information, making them suited for clinical adoption without the necessity of modifying existing systems. However, the lack of the other input sources could limit generalizability with reduced estimation accuracy when visually similar yet different anatomies are concerned. Furthermore, the methodological decision to use monocular vision instead of stereo vision makes scene understanding more challenging, as it lacks access to absolute depth maps and surface reconstructions—the effect of all of which would be meaningful future studies.

While models presented in this work show promising results, further investigation could proceed by adding additional model knowledge with the inclusion of a local depth-vision keypoint tracking module. Furthermore, the motion in the dataset is restricted to retraction, which could confine the model to this task. It is therefore meaningful to continue exploring how networks will perform in *ex vivo* and *in vivo* environments by collecting more surgical datasets [30]. It should be noted that any clinical feedback implementation will only be possible after further validation with *in vivo* data, following which could a response platform be built to provide assessments in training or to enable direct intraoperative haptic feedback. Furthermore, though recorded inference times imply real-time model capabilities, it is important to note that actual deployment is affected by delays in video frame acquisition, buffering, and overhead times of the systems that make such performance an upper bound rather than a guarantee.
